# Effects of partial soybean meal replacement with sunflower meal and non-starch polysaccharide degrading enzymes supplementation on broiler growth performance, nutrient digestibility, and gut morphology

**DOI:** 10.14202/vetworld.2025.695-704

**Published:** 2025-03-23

**Authors:** Zeeshan Munawar, Saad Amjid, Faisal Ramzan, Azhar Rafique, Safdar Hassan, Urooj Anwar, Momna Mehmood, Umar Farooq, Muhammad Farooq Khalid, Riaz Mustafa, Muhammad Riaz, Muhammad Aziz ur Rahman, Waseem Abbas

**Affiliations:** 1 Institute of Animal and Dairy Sciences, University of Agriculture, Faisalabad, Pakistan; 2 Department of Zoology, Government College University, Faisalabad, 38000, Pakistan; 3 Department of Animal and Dairy Science Muhammad Nawaz Shareef University of Agriculture, Multan, 66000, Pakistan; 4 Sub Campus, Toba Tek Sing, University of Agriculture, Faisalabad, 36050, Pakistan

**Keywords:** broiler nutrition, feed efficiency, gut morphology, non-starch polysaccharide degrading enzymes, nutrient digestibility, soybean meal replacement, sunflower meal

## Abstract

**Background and Aim:**

Soybean meal (SBM) is the primary protein source in broiler diets; however, its high cost and import dependency necessitate alternative protein sources. Sunflower meal (SFM) is a viable alternative but contains high fiber and non-starch polysaccharides (NSPs), which can impair nutrient utilization. This study evaluated the effects of partially replacing SBM with SFM, with or without NSP degrading enzymes (NSPase) enzyme supplementation, on growth performance, nutrient digestibility, digesta viscosity, and gut morphology in broilers.

**Materials and Methods:**

A total of 588 day-old Ross-308 broiler chicks were randomly assigned to six dietary treatments following a 3 × 2 factorial design, with three levels of SBM replacement (0%, 10%, and 20% SFM) and two levels of NSPase supplementation (0 or 100 g/ton feed). Diets were formulated for the starter (1–10 days), grower (11–21 days), and finisher (22–35 days) phases. Growth performance (body weight gain [BWG], feed intake [FI], feed conversion ratio [FCR]), nutrient digestibility (dry matter, crude protein, ether extract, and crude fiber [CF]), digesta viscosity, and gut morphology (villus height [VH], crypt depth [CD], villus width, and villus-to-crypt ratio) were assessed. Statistical analysis was conducted using two-way analysis of variance with Tukey’s test for mean comparisons (p < 0.05).

**Results:**

Replacing SBM with up to 20% SFM did not significantly impact BWG (p > 0.05), FI (p > 0.05), or FCR (p > 0.05). However, digesta viscosity increased significantly with higher SFM levels (p < 0.001), while NSPase supplementation reduced viscosity (p < 0.001). CF digestibility was lower with increasing SFM levels (p < 0.01) but improved with NSPase addition (p < 0.01). Gut morphology parameters, including VH and CD, were negatively affected by higher SFM inclusion but showed improvement with NSPase supplementation.

**Conclusion:**

SBM can be partially replaced with up to 20% SFM in broiler diets without compromising growth performance. However, increasing SFM levels can reduce CF digestibility and increase digesta viscosity. The addition of NSPase enzymes mitigates these adverse effects by enhancing fiber digestibility and reducing gut viscosity. These findings support the use of SFM as an economically viable protein alternative in broiler feed formulations, particularly in SBM-importing regions.

## INTRODUCTION

Broiler requires high-quality protein, energy, vitamins, and minerals for optimum growth. Animal protein sources for a broiler diet are not preferred due to the risk of contamination and the varying amount of available protein [1]. Plant-based protein sources are widely used, and soybean meal (SBM) is preferred due to its high-quality protein and well-balanced amino acid profile [2]. However, changes in the price of SBM directly influence the poultry industry, particularly when imported SBM is used in the broiler diet [1]. Therefore, protein sources like sunflower (*Helianthus annuus*) meal (SFM) could be a potential alternative to SBM in the broiler diet [3, 4]. However, the use of SFM in the broiler diet has been restricted due to the high fiber content and low level of energy and lysine [4].

Sunflower seed typically contains 35%–40% hulls and 60%–65% protein core, and sunflower hulls contain approximately 50% cellulose and 25% lignin. Sunflower meal (SFM) is prepared from sunflower seeds after oil extraction using a solvent and it contains 30%–34% protein, 4.6% soluble, and 22% insoluble non-starch polysaccharides (NSPs). The NSPs of SFM comprised 41% cellulose, 25% pectin polysaccharides, 24% 4-Omethl-glucuronoxylans, 4.5% galactomannans, and 5% fucoxyloglucans. The presence of NSPs in broiler diets decreases the performance of broiler chickens by decreasing the uptake of nutrients due to increased digesta viscosity, thereby decreasing feed efficiency [5]. NSP-degrading enzymes (NSPase) can be used to reduce NSPs in SFM [5]. The use of exogenous NSPase enzymes increases the digestion of undigested portions of the diet by reducing digesta viscosity [6]. Furthermore, exogenous NSPase enzymes provide an additional calorific benefit by releasing energy through the breakdown of undigested feed components [7, 8]. Studies have examined the performance of broiler chicks fed different levels of SFM as a partial replacement for standard protein sources such as SBM [3, 9, 10]. Attia *et al*. [3] reported that SFM could be included at up to 15% in the growing and finishing diets of broilers without affecting growth performance. DMaria de Moraes Oliveira *et al*. [9] incorporated SFM up to 16% levels in broiler diets from day 1 to 21. Their findings indicated that adding SFM up to 8% did not affect growth performance; however, the addition of 16% significantly affected performance. Furthermore, the addition of a multienzyme complex improved growth performance [9]. Additional research evaluated the effects of the sunflower cake and enzyme complex on broiler performance. The study incorporated up to 20% sunflower cake and concluded that sunflower cake with an enzymatic complex could be effectively used up to 10% in broiler feeding from 21 to 42 days of age [10]. Yaqoob *et al*. [11] found no adverse effects from including sunflower seed meal at levels of up to 3%, 6%, and 9%, respectively, in broiler diets.

While SBM remains the predominant protein source in broiler diets due to its superior amino acid profile, its high cost and dependence on imports have necessitated the exploration of alternative plant-based protein sources. SFM presents a viable alternative, offering a moderate protein content. However, its high fiber and NSP content hinder nutrient digestibility and increase digesta viscosity, which can negatively impact broiler growth performance and gut health. Prior research has examined the inclusion of SFM in broiler diets, but findings on its optimal replacement levels and the role of NSPase enzymes in mitigating associated drawbacks remain inconsistent. Some studies have indicated that SFM can replace SBM up to 15%–16% without adverse effects, while others suggest that higher inclusion rates impair performance. In addition, the potential of NSPase enzymes in counteracting the negative effects of high SFM inclusion requires further elucidation.

This study aims to evaluate the effects of partially replacing SBM with SFM (at 10% and 20% inclusion levels) in broiler diets, with and without NSPase enzyme supplementation. The research specifically investigates growth performance, nutrient digestibility, digesta viscosity, and gut morphology to determine the feasibility of utilizing SFM as a cost-effective alternative protein source in poultry nutrition while mitigating its limitations through enzymatic supplementation.

## MATERIALS AND METHODS

### Ethical approval

The study received ethical approval for animal experimentation from the Institute of Animal and Dairy Sciences, University of Agriculture, Faisalabad, Pakistan (No. DGS/19697-700).

### Study period and location

The experiment was conducted for 35 days during November and December 2021 at Dr. Raja Muhammad Akram Animal Nutrition Research Center in University of Agriculture, Faisalabad.

### Animal housing and experimental design

Five hundred and eighty-eight (588)-day-old Ross-308 chicks were randomly allocated into six treatment groups with seven replicates (experimental units/pens) of 14 chicks each. On arrival, the birds were given a sugar solution (250 g/L) to alleviate transportation stress. Throughout the trial period, the birds in each treatment were fed experimental diets *ad libitum*. Vaccinations were administered according to recommended guidelines for Ross-308 broilers.

### Experimental diets

Six iso-nitrogenous and isocaloric experimental diets were formulated for the starter (days 1–10), grower (days 11–21), and finisher (days 22–35) phases (Table 1). The control diet (CONTROL) was formulated without SFM and with or without NSPase enzyme supplementation The experimental diets contained 10% (SFM10) or 20% (SFM20) SFM, each formulated with or without NSPase. NSPase was added at 100 g/ton of feed per the manufacturer’s recommendations (Suntaq International Limited, China). Winzyme MXP served as an enzyme source of xylanase (12,000 IU/kg), mannanase (3200 IU/kg), and protease (80000 IU/kg). The complete dietary compositions are presented in Table 1. Throughout the trial, the birds had *ad libitum* access to both feed and water.

**Table 1:** Stater, grower, and finisher diet ingredient composition.

Ingredients (%)	Starter			Grower			Finisher		
		
	Control	SFM 10%	SFM 20%	Control	SFM 10%	SFM 20%	Control	SFM 10%	SFM 20%
Corn	50.62	49.39	47.81	52.85	51.83	50.54	56.94	55.69	54.36
Soybean meal	37.66	31.76	25.91	34.33	28.39	22.49	30.57	24.66	18.75
Sunflower meal	0	10	20	0	10	20	0	10.00	20.00
Rice hulls	6.63	3.21	0	6.92	3.38	0	6.78	3.38	0.00
Soybean oil	1.81	2.32	2.92	3.1	3.55	4.08	3.31	3.82	4.39
Calcium carbonate	1.08	1.06	1.04	1.06	1.04	1.06	0.96	0.94	0.98
Monocalcium phosphate	0.68	0.65	0.62	0.47	0.44	0.41	0.35	0.35	0.35
Sodium chloride	0.27	0.26	0.24	0.28	0.26	0.24	0.27	0.25	0.24
Sodium bicarbonate	0.39	0.42	0.44	0.39	0.42	0.45	0.40	0.42	0.45
Lysine sulphate55%	0.29	0.40	0.50	0.20	0.31	0.42	0.12	0.23	0.34
DL-Methionine	0.32	0.29	0.25	0.25	0.22	0.18	0.19	0.16	0.12
L-Threonine, 99%	0.09	0.1	0.11	0.05	0.07	0.08	0.01	0.02	0.03
Valine	0.05	0.04	0.04	0	0	0	0.05	0.05	0.05
Vitalink	0.05	0.05	0.05	0.05	0.05	0.05	0.05	0.05	0.05
Nutrimin	0.05	0.05	0.05	0.05	0.05	0.05	0.05	0.05	0.05
Winzyme MXP	0	0	0	0	0	0	0	0	0
Winzyme HTR	0.005	0.005	0.005	0.005	0.005	0.005	100	100	100
Calculated composition									
ME (Kcal/kg)	2850	2850	2850	2950	2950	2950	3050	3050	3050
CP (%)	22	22	22	20.5	20.5	20.5	19	19	19
EE (%)	4.40	4.82	5.39	5.58	6.03	6.53	5.12	5.23	5.33
CF (%)	5.57	5.50	5.57	5.50	5.45	5.46	6.71	7.20	7.70
Ca (%)	0.85	0.85	0.85	0.80	0.80	0.80	0.75	0.75	0.75
P, available (%)	0.45	0.45	0.45	0.40	0.40	0.40	0.39	0.39	0.39
Na (%)	0.23	0.23	0.23	0.23	0.23	0.23	0.23	0.23	0.23
Cl (%)	0.23	0.23	0.23	0.23	0.23	0.23	0.23	0.23	0.23
Lys. Dig (%)	1.23	1.23	1.23	1.10	1.10	1.10	0.97	0.97	0.97
Met. Dig (%)	0.62	0.61	0.60	0.53	0.52	0.51	0.46	0.45	0.44
Methionine + Cysteine dig (%)	0.89	0.89	0.89	0.79	0.79	0.79	0.70	0.70	0.70
Threonine dig (%)	0.82	0.82	0.82	0.74	0.74	0.74	0.65	0.65	0.65
Tryptophan dig (%)	0.25	0.24	0.24	0.23	0.22	0.22	0.21	0.21	0.20
Arginine dig (%)	1.37	1.43	1.48	1.28	1.33	1.39	1.18	1.23	1.29
Leucine dig (%)	1.65	1.62	1.59	1.57	1.54	1.51	1.48	1.59	1.43
Isoleucine dig (%)	0.84	0.82	0.81	0.78	0.77	0.75	0.72	0.70	0.69
Valine dig (%)	0.95	0.95	0.95	0.84	0.85	0.85	0.78	0.71	0.79

Each kilogram of Vitalink® provided: 5400 KIU of Vitamin D3, 20 mg of Vitamin B12, 1600 mg of folic acid, 4000 mg of Vitamin K3, 9000 mg of Vitamin B2, 20000 KIU of Vitamin A, 4000 mg of Vitamin B1, 200 mg of biotin, 48000 mg of Vitamin E, 7600 mg of Vitamin B6, and 60000 mg of niacin, along with 20000 mg of pantothenic acid. Additionally, each kilogram of Nutrimin® supplied: 400 mg of cobalt, 140000 mg of manganese, 12000 mg of copper, 120000 mg of zinc, 1800 mg of iodine, 10000 mg of iron, and 360 mg of selenium. CF=Crude fiber, EE=Ether extract, CP=Crude protein, Ca=Calcium, P=Phosphorus, NSP=Non-starch polysaccharides, SFM=Sunflower meal, ME=Metabolizable energy, dig=Digestible, Wnzyme MXP and Winzyme HTR are the products from Suntaq International Limited, China

### Growth performance

The initial body weight was recorded on day 1, followed by weekly measurements of body weight and feed intake (FI). The recorded data were used to calculate weekly weight gain and FI using the following equation:


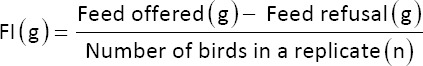


The feed conversion ratio (FCR) was calculated weekly to determine the feed efficiency. It was calculated using the following equation:


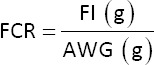


Mortality was monitored throughout the trial period by recording the numbers and weights of the dead birds. The mortality data were used to calculate the corrected FI and FCR.

### Nutrient digestibility

Nutrient digestibility was determined using an external marker method on days 21 and 35 of the trial. Celite^®^ (Diatomite product manufactured by Imerys S.A, Paris) was added to the experimental diets at a concentration of 1% before fecal collection. Birds were given a 3-day adoption period before sampling. Fecal samples were collected over 24 h on both days 21 and 35 by placing plastic sheets under each pen. The collected fecal samples were stored in labeled zipper bags by replicating at −10°C until analysis for acid-insoluble ash (AIA). Experimental diets and fecal samples were analyzed for AIA and nutrient composition following the Association of Official Analytical Chemists International (AOAC) (2005) method, as described by Aziz ur Rahman *et al*. [7]. The coefficient of digestibility of each nutrient was calculated using the following equation:


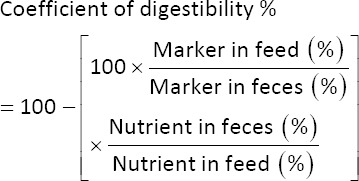


### Gut Morphometry

On days 21 and 35, one bird from each replicate was selected for tissue sampling. A section of the jejunum was excised and initially washed with neutral buffered formaldehyde. The tissue samples were then stored in 10% formalin solution for later use. The preserved samples were dehydrated using increasing alcohol concentrations, followed by clearing with xylene (Table 2).

**Table 2:** Dehydration of gut samples with alcohol and xylene.

Alcohol concentration (%)	Time span
70	Overnight
80	60 min
95	60 min
95	60 min
100	90 min
100	90 min
Xylene + Alcohol I	90 min
Xylene I	60 min
Xylene II	60 min

After dehydration and clearing, tissue samples were embedded in paraffin to provide structural support. Two 1-L beakers of paraffin were placed in an oven at 60°C. The tissues underwent a two-stage paraffin infiltration process: First, in paraffin I for 2 h, followed by overnight infiltration in paraffin II. Tissues were then embedded in molten paraffin wax to form tissue blocks using a dispenser. Each fixed tissue block was sectioned to 5–7 μm thickness using a microtome. Sectioned pieces were placed in a water bath for mounting. Clean glass slides were prepared by applying a thin layer of egg albumin. Tissue samples were carefully mounted onto slides from a water bath. The mounted sections were stained with hematoxylin and eosin protocol. Villus length, width, and crypt depth (CD) measurements were performed using J-image software version 1.54 (https://imagej.en.lo4d.com/windows) [12].

### Digesta viscosity

The digesta viscosity of the intestinal contents collected after slaughtering was measured using a Brookfield DV-E viscometer (AMETEK Brookfield, USA). The samples were first centrifuged for 10 min and then analyzed using a Brookfield DV-E rotatory viscometer (AMETEK Brookfield) with spindle number four [13].

### Chemical analysis

All experimental diets and composite fecal samples were chemically analyzed for dry matter (DM), crude fiber (CF), ether extract (EE), and crude protein (CP) according to the AOAC (2005) protocol, as described by Aziz ur Rahman *et al*. [7]. The AIA content of the diet and dried composite fecal samples was determined using the method described by De Coca-Sinova *et al*. [14].

### Statistical analysis

All data were analyzed using a two-way analysis of variance (ANOVA) to evaluate the main effects of SBM replacement with SFM at three levels (0%, 10%, and 20%) and NSPase enzyme supplementation (with vs. without), as well as their interaction effects on broiler growth performance, nutrient digestibility, digesta viscosity, and gut morphology. The statistical model included SFM level and NSPase supplementation as fixed factors, with replicate pens serving as the experimental unit. *Post hoc* comparisons were conducted using Tukey’s Honestly Significant Difference test to identify significant differences among treatment means when ANOVA results indicated statistical significance (p < 0.05). All statistical analyses were conducted using R software version 4.2.3 (https://cran.r-project.org).

## RESULTS

### Growth performance

The replacement of SBM with 10% or 20% SFM in the diet did not significantly impact body weight gain (BWG) or FI during the grower and finisher phases (p > 0.05). However, the FCR was significantly lower (p = 0.04) in the control diet (without SFM) during the grower phase, although it did not differ significantly from the other diets during the finisher phase (Table 3). The BWG in the Control and SFM10 groups (901.1 g and 893.3 g, respectively) did not differ significantly from that in the SFM20 group during the growing phase (p = 0.14). Similarly, no significant differences were observed in BWG during the finishing phase (1304.7 g and 1293.5 g for control and SFM10, respectively; p = 0.73) compared with the SFM20 group.

**Table 3:** Effects of partially replacing Soybean meal with SFM and NSPase enzyme on growth performance.

SFM (%)	ENZ	Days 1–21			Days 22–35			Days 1–35		
		
		BWG (g)	FI (g)	FCR	BWG (g)	FI (g)	FCR	BWG (g)	FI (g)	FCR
0		901.1	1153.9	1.28^a^	1304.7	2168.7	1.66	2205.8	3322.6	1.47
10		893.3	1156.8	1.30^ab^	1293.5	2099.5	1.64	2186.8	3256.3	1.47
20		864.1	1149.5	1.33^b^	1273.5	2142.9	1.68	2137.6	3292.4	1.51
p-value		0.14	0.94	0.04	0.73	0.34	0.49	0.13	0.40	0.15
	No	879.1	1151.0	1.31	1255.1	2103.7	1.68	2134.2	3254.7	1.49
	Yes	893.2	1155.8	1.30	1325.9	2170.4	1.64	2219.1	3326.2	1.47
p-value		0.37	0.78	0.38	0.04	0.09	0.36	0.004	0.08	0.21
0	No	884.7	1150.5	1.30	1268.9	2094.6	1.65	2153.6	3245.1	1.48
	Yes	917.6	1157.2	1.26	1340.4	2242.7	1.67	2258	3399.9	1.46
10	No	886.6	1159.7	1.31	1245.0	2069.9	1.67	2131.6	3229.6	1.49
	Yes	900.1	1153.9	1.28	1342.1	2129.2	1.59	2242.2	3283.1	1.44
20	No	866.2	1142.8	1.32	1251.3	2146.7	1.71	2117.5	3289.5	1.52
	Yes	862.1	1156.3	1.34	1295.2	2139.2	1.66	2157.3	3295.5	1.5
p-value		0.63	0.90	0.26	0.80	0.26	0.51	0.51	0.30	0.59

The table presents the mean values of various variables, with superscript letters indicating that differences between the means are statistically significant (p < 0.05). SFM 0%=Without SFM; SFM 10%=The added level of SFM was 10%; SFM 20%=The added level of SFM was 20%; Diets were offered either with (100 g/ton of feed) or without NSPase Enzyme. ENZ=NSPase enzymes, BWG=Body weight gain, FI=Feed intake, FCR=Feed conversion ratio, NSP=Non-starch polysaccharides, SFM=Sunflower meal

The addition of the NSPase enzyme significantly improved BWG during the finishing phase (p = 0.04) and for the overall period (1–35 days) (p = 0.004). On 35 days of age, the group without the NSPase enzyme had an average final weight of 2134.2 g, whereas the group supplemented with the NSPase enzyme had an average final weight of 2219.1 g.

The combined effect of SBM replacement with SFM and NSPase supplementation did not significantly affect BWG (p = 0.51), FI (p = 0.30), and FCR (p = 0.59) in broilers. Among all the treatments, the control group with NSPase enzyme exhibited the highest final body weight (2258 g), whereas the SFM10 group with NSPase enzyme demonstrated the lowest FCR (1.44).

### Digesta viscosity

The increase in dietary SFM levels significantly increased digesta viscosity at both 21 (p = 0.001) and 35 (p = 0.001) days of age. In addition, the addition of the NSPase enzyme significantly reduced digesta viscosity at both 21 (p = 0.001) and 35 (p = 0.001) days of age. The interactions between SFM levels and NSPase enzyme inclusion in the diet significantly (p < 0.03) affected digesta viscosity. The control diet supplemented with the NSPase enzyme showed the lowest digesta viscosity on 21 and 35 days (2.69 cps and 2.55 cps, respectively), whereas the diet containing 20% SFM without enzyme supplementation had the highest digesta viscosity (5.14 cps and 4.76 cps, respectively) compared with the other dietary treatments (Table 4).

**Table 4:** Effect of partially replacing Soybean meal with SFM and NSPase enzyme on digesta viscosity in broiler on days 21 and 35.

SFM (%)	ENZ	Days 21	Days 35
	
		Digesta viscosity (cps)	Digesta viscosity (cps)
0		3.04^a^	2.83^a^
10		3.53^b^	3.25^b^
20		4.77^c^	4.41^c^
p-value		0.001	0.001
	No	4.15	3.82
	Yes	3.41	3.17
p-value		0.001	0.001
0	No	3.40^a^	3.11^a^
	Yes	2.69^b^	2.55^b^
10	No	3.91^c^	3.60^c^
	Yes	3.16^a^	2.91^ab^
20	No	5.14^d^	4.76^d^
	Yes	4.39^e^	4.06^e^
p-value		0.01	0.02

The table presents the mean values of various variables, with superscript letters indicating that differences between the means are statistically significant (p < 0.05). SFM0%=Without SFM; SFM10%=The added level of SFM was 10%; SFM20%=The added level of SFM was 20%; Diets were offered either with (100 g/ton of feed) or without NSPase Enzyme, NSP=Non-starch polysaccharides, ENZ=NSPase enzymes, SFM=Sunflower meal

### Gut Morphology and nutrient digestibility

Higher dietary SFM levels significantly reduced villus height (VH), CD, and the villus-to-crypt ratio (villus: crypt) on days 21 and 35 (Table 5). The increased SFM levels also lead to a significant increase in villus width (VW) on day 21 (p = 0.02), although no significant differences were noted on day 35 (p = 0.58). The addition of NSPase at 100 g/ton of feed significantly reduced VH and CD on days 21 and 35. Similarly, NSPase enzyme supplementation significantly decreased VW on day 21, but this effect was not observed on day 35. The interaction between SFM inclusion and enzyme supplementation had no significant effect on gut morphology (Table 5).

**Table 5:** Effects of partially replacing soybean meal with SFM and NSPase enzyme on intestinal morphology in broiler on days 21 and 35.

SFM (%)	ENZ	Days 1–21				Days 22–35			
	
		VH (µm)	CD (µm)	VW (µm)	Villus: Crypt	VH (µm)	CD (µm)	VW (µm)	Villus: Crypt
0		526.24^a^	108.14^a^	64.45^a^	4.87^a^	742^a^	142.13^a^	102.64	5.22^a^
10		498.69^b^	104.34^b^	65.50^a^	4.78^a^	716^b^	137.98^b^	102.48	5.19^a^
20		485.26^c^	105.30^b^	67.91^b^	4.61^b^	701^b^	140.34^ab^	97.01	4.44^b^
p-value		0.001	0.01	0.02	0.01	0.01	0.03	0.58	0.01
	No	511.63^a^	107.22^a^	67.20^a^	4.77	729.31^a^	141.11^a^	98.18	5.17
	Yes	495.16^b^	104.63^b^	64.70^b^	4.73	709.74^b^	139.19^b^	103.24	5.10^a^
p-value		0.01	0.02	0.01	0.16	0.01	0.03	0.32	0.12
0	No	537.67	109.20	66.06	4.93	753.71	143.05	102.67	5.27
	Yes	514.81	107.09	62.84	4.81	729.97	141.22	102.60	5.17
10	No	505.13	105.36	66.84	4.80	725.24	138.67	102.26	5.23
	Yes	492.24	103.31	64.16	4.77	706.76	137.30	102.70	5.15
20	No	492.10	107.10	68.71	4.60	708.99	141.62	89.60	5.0
	Yes	478.41	103.50	67.11	4.62	692.50	139.05	104.41	4.98^c^
p-value		0.29	0.63	0.64	0.21	0.85	0.84	0.40	0.67

The table presents the mean values of various variables, with superscript letters indicating that differences between the means are statistically significant (p < 0.05). SFM 0%=Without SFM; SFM 10%=The added level of SFM was 10%; SFM 20%=The added level of SFM was 20%. ENZ=NSPase enzymes. Diets were offered either with (100 g/ton of feed) or without NSPase Enzyme, NSP=Non-starch polysaccharides, SFM=Sunflower meal, VH=Villus height, VW=Villus width, CD=Crypt depth

The incorporation of dietary SFM reduced CF digestibility at both 21 (p = 0.001) and 35 (p = 0.004) days of age (Table 6). NSPase enzyme supplementation improved DM digestibility on day 21 and improved both CP and CF digestibility on days 21 (p < 0.05) and 35 (p < 0.05). A significant interaction between SFM inclusion and NSPase enzyme supplementation was observed for CF digestibility on day 21 (p = 0.03). Specifically, both the control and SFM10 groups supplemented with NSPase enzyme exhibited the highest CF digestibility (21%), whereas the SFM20 group without enzyme supplementation showed the lowest CF digestibility (17%). However, the combined effect of SFM and NSPase enzyme did not affect the digestibility of DM, EE, and CP on days 21 and 35 (Table 6).

**Table 6:** Effects of partially replacing Soybean meal with SFM and NSPase enzyme on nutrient digestibility in broiler on days 21 and 35.

SFM (%)	ENZ	Day 21				Day 35			
		DM (%)	CP (%)	EE (%)	CF (%)	DM (%)	CP (%)	EE (%)	CF (%)
0		78.13	70.57	80.29	20.22^a^	79.23	71.50	80.79	20.49^a^
10		79.47	70.64	80.64	19.96^a^	79.30	70.57	79.36	20.78^a^
20		79.94	69.14	79.00	17.89^b^	79.81	70.00	81.79	18.76^b^
p-value		0.31	0.08	0.37	0.001	0.60	0.17	0.40	0.004
	No	78.16	69.10	80.29	18.42	78.73	69.76	80.57	19.18
	Yes	80.20	71.14	79.67	20.29	80.16	71.62	80.71	20.85
p-value		0.04	0.04	0.32	0.001	0.11	0.04	0.88	0.001
0	No	77.06	69.57	80.29^ab^	19.29^b^	78.69	71.00	80.71	19.55^b^
	Yes	79.20	71.57	80.29^ab^	21.14^a^	79.77	72.00	80.86	21.44^a^
10	No	78.60	69.14	82.14^a^	18.89^bc^	78.41	69.71	79.14	19.62^bc^
	Yes	80.34	72.14	79.14^ab^	21.03^a^	80.18	71.43	79.57	21.94^a^
20	No	78.83	68.57	78.43^b^	17.09^c^	79.08	68.57	81.86	18.36^c^
	Yes	81.06	69.71	79.57^ab^	18.69^bc^	80.54	71.43	81.71	19.16^bc^
p-value		0.98	0.74	0.37	0.03	0.86	0.40	0.91	0.04

The table presents the mean values of various variables, with superscript letters indicating that differences between the means are statistically significant (p < 0.05). SFM0%=Without SFM; SFM 10%=Added level of SFM was 10%; SFM 20%=Added level of SFM was 20%. Diets were offered either with (100 g/ton of feed) or without NSPase Enzyme. ENZ=NSPase enzyme, DM=Dry matter, CP=Crude protein, EE=Ether extract, CF=Crude fiber, NSP=Non-starch polysaccharides, SFM=Sunflower meal

## DISCUSSION

SBM is extensively used as a protein source in poultry feed [15, 16]. Many countries rely on imports for soybean supply, making them vulnerable to fluctuations in the international market caused by supply chain disruptions or political events [1]. These fluctuations exert substantial pressure on the local markets of importing nations [17]. Developing countries are particularly susceptible to these challenges, and the need to import SBM further strains their already struggling economies [18, 19]. Considering the need for an alternative protein source, we designed the present study to evaluate the effect of partially replacing SBM with SFM in broiler diets.

The findings of this study indicate that replacing SBM with SFM at levels up to 20%, supplemented with 100 g/ton of NSPase enzyme, does not adversely affect BWG and FI in broilers. These results align with Bilal *et al*. [20], who added SFM with or without the NSPase enzyme (Zympex 008^®^, Impextraco, Belgium) and similarly found no significant improvement in BWG and FI among the different treatments. Likewise, Yaqoob *et al*. [11] replaced SBM with 3%, 6%, and 9% SFM combined with multienzymes (Axtra XAP 101, Chemunique, South Africa) and observed no significant differences between the treatment groups. In contrast, other studies have documented that substituting SBM with SFM up to 16% can negatively impact growth performance and FI in broilers up to 21 days of age. This adverse effect has been attributed to the high fiber and NSP levels in SFM [9]. Similarly, Kyrkelanov *et al*. [21] reported a reduction in the body weight of broilers after replacing the SBM with SFM.

Berwanger *et al*. [10] reported that adding more than 10% sunflower cake to broiler diets adversely affected growth performance from day 21 to day 42. They attributed this adverse effect to an increase in digesta viscosity due to the high levels of NSPs in the diet. Although our study did not find any significant effect on FI due to the addition of SFM, a numerical decrease in FI was noted at higher SFM levels. This observation is similar to other studies that reported decreased FI with the inclusion of sunflower cake [10]. In addition to growth performance, Yalçin *et al*. [22] have reported that replacing SBM with SFM did not affect the pH or color of chicken breast.

Previous studies by DMaria de Moraes Oliveira *et al*. [9], Yaqoob *et al*. [11], Bilal *et al*. [20], Kyrkelanov *et al*. [21] have reported inconsistent responses of SFM to broiler performance. These differing results can be attributed to the quality of SFM, as well as its higher levels of NSPs and fiber content, which seem to be limiting factors in poultry diets. The fiber content in broiler diets affects the physical density of the feed; higher fiber content increases the volume occupied by fiber in the digestive tract, thereby reducing FI. This can also interfere with the water retention capacity of the diet. SFM is known for its low energy and lysine content; a deficiency in both can severely impact the growth performance of broiler. In this study, we supplemented all diets with lysine and energy sources to meet the nutritional requirements of broiler birds. These supplementations likely explain why we did not observe any adverse effects on growth performance despite the inclusion of 20% SFM in the diets.

Our results indicate a significantly poorer FCR (p = 0.04) when SFM was included in the broiler diet from day 1 to day 21. However, this difference disappears during the finishing phase. Similarly, DMaria de Moraes Oliveira *et al*. [9] reported a poor FCR when the SBM was replaced with SFM up to 21 days of age. Furthermore, Hong *et al*. [23] also reported a poor FCR when 15% SFM was added. Poor FCR due to higher SFM clearly reflects increased dietary CF. The adverse effects of high SFM inclusion were primarily observed during the starter phase but diminished during the finishing phase, likely because older birds have a more developed digestive system. As broilers mature, their capacity to handle dietary fiber improves due to enhanced digestive enzyme production and a more robust gut microbiome. In addition, older birds typically demonstrate better tolerance to anti-nutrient factors present in SFM, allowing them to maintain more efficient nutrient use despite higher fiber content. Although NSPase did not significantly affect the FCR, a numerical improvement was observed when the enzymes were added. Other studies have also found that adding enzymes more evidently improves the FCR during the grower phase compared to the finishing phase [24, 25]. The effect of enzymes during the growing phase might be due to the young age of broiler birds, as their digestive tract is not yet at its maximum capacity, which may explain these differences.

Similar to Yaqoob *et al*. [11], we did not observe any difference in DM digestibility with increasing SFM. In this study, the addition of SFM significantly reduced the digestibility of CF on days 21 and 35. Other studies have reported that up to 9% SFM has no effect on CF digestibility [11]. The digestibility of DM, CP, and CF was improved by the addition of NSPase. Similarly, studies have reported significantly better DM digestibility [23, 26] and CP digestibility [11, 27] with the addition of enzymes. The improved CF digestibility observed in our study is attributed to NSPase enzymes facilitating the digestion of CF in the diet [26, 28]. The replacement of SBM with SFM at 10% and 20% increased digesta viscosity, whereas the addition of NSPase enzymes significantly reduced digesta viscosity on 21 and 35 days of age. Similarly, Horvatovic *et al*. [29] reported an increase in digesta viscosity due to SFM, which was mitigated by the addition of enzymes. The inclusion of NSPase enzymes not only reduced viscosity but also improved the digestibility of CF and, ultimately, BWG during the finishing phase. The addition of exogenous enzymes enhanced overall digestibility by increasing the activity of digestive enzymes due to greater substrate availability. Furthermore, the addition of amylase and protease enhances pancreatic and intestinal enzyme activity [30].

The development of VH occurs primarily during the first 10 days after hatching [31]. The inclusion of 20% SFM in the diet reduced VH, VW, and CD on days 21 and 35. Intestinal morphology is a critical indicator of gut health, and anti-nutrient and other stressors can adversely affect the intestinal mucosa. Shortened VH is associated with the presence of toxins [32]. The increased fiber content in SFM can cause physical irritation to the developing intestinal mucosa. This mechanical stress may trigger protective mechanisms in the intestinal tissue, such as reducing villus size to minimize surface exposure to potential irritants. The higher digesta viscosity caused by NSPs in SFM can form a barrier between nutrients and the intestinal surface, reducing the stimulatory effect that nutrient absorption typically has on villus development. Furthermore, anti-nutrient factors in SFM, particularly chlorogenic acid, may directly affect intestinal cell proliferation and turnover. These compounds can increase oxidative stress and inflammation in the intestinal tissue, potentially leading to reduced VH and width. The decreased CD suggests compromised cellular regeneration capacity because the crypts are the primary site of new enterocyte production.

Consistent with our findings, Berwanger *et al*. [10] reported a reduction in VH with increased inclusion levels of sunflower cake; however, contrary to our results, they also observed an increase in CD. Moghaddam *et al*. [33] reported a quadratic response for VH and CD when SBM was replaced with SFM, and they reported a shorting of VH and an increase in CD when 21% SFM was used in the broiler diet. Our study found that the addition of NSPase significantly reduced VH and CD (p = 0.001). The addition of NSPase might have created an environment in which the gut did not need to maintain long villi for nutrient absorption, leading to a more metabolically efficient but shorter villus structure. Similarly, Berwanger *et al*. [10] reported an increase in duodenal VH without enzyme complex supplementation. However, these findings contrast those of DMaria de Moraes Oliveira *et al*. [9] and Yaqoob *et al*. [11], who reported a significant increase in VH with the addition of multienzymes.

The results showed that a higher SFM level increased the VW during the starter phase, whereas this effect was diminished during the finishing phase. The increase in VW on day 21 with higher SFM levels could be due to an initial adaptive response of the intestinal mucosa to handle the higher fiber content and anti-nutritional factors present in SFM. The intestine typically responds to dietary changes by modifying its morphology to optimize nutrient absorption. By day 35, the lack of significant differences may indicate that the birds had fully adapted to the diet. Older birds generally have a more developed digestive system and a better capacity to handle fibrous feedstuffs, which could explain why these differences have disappeared. Similarly, Moghaddam *et al*. [33] reported that adding 21% SFM resulted higher VW compared with adding 7% or 14% SFM on 28 days of age.

In this study, NSPase supplementation decreased VW on day 21, but this effect was not observed on day 35. The initial decrease in VW with enzyme supplementation might result from the rapid breakdown of NSPs, reducing the physical stimulation on the intestinal wall. NSPase enzymes break down complex fiber structures, potentially decreasing the mechanical stress that typically promotes villus development. In addition, the faster digestion of NSPs may temporarily reduce the substrate availability for beneficial gut bacteria, affecting the production of metabolites that support villus development. The disappearance of enzyme effects by day 35 suggests that the birds’ digestive system has achieved a new equilibrium. As birds age, their endogenous enzyme production and gut microbiota become more established, potentially decreasing their dependence on exogenous enzyme supplementation. This maturation process may explain why the initial differences in VW were no longer apparent.

Our results showed a linear decrease in the VH:CD ratio with increasing SFM inclusion. These observations were aligned with Yaqoob *et al*.’s [11] findings, which noted a decrease in the VH:CD ratio with 9% SFM inclusion. Conversely, Attia *et al*. [3] observed a quadratic response to SFM, identifying the lowest ratio at a 21% SFM inclusion level. Higher fiber content in SFM can affect the epithelial and mucin layers, potentially damaging the intestinal wall and villus apex [34]. Higher VH indicates improved nutrient absorption due to increased surface area, whereas crypts are sites of cell division that contribute to villus renewal. VW and CD of birds fed the SBM-based diet were worse than those fed the SFM diet. Although our study observed a reduction in VH, VW either increased with SFM inclusion until day 21 or remained unaffected on day 35. Studies have reported that anti-nutritional factors in SBM affect gut morphology and functions in animals [35], which could explain the better VW and CD in birds fed SFM.

## CONCLUSION

This study demonstrated that SBM can be partially replaced with up to 20% SFM in broiler diets without adversely affecting growth performance and FI during the grower and finisher phases. However, increasing SFM levels significantly increased digesta viscosity (p < 0.001) and reduced CF digestibility (p = 0.004). The inclusion of NSPase enzymes (100 g/ton) effectively mitigated these adverse effects by improving nutrient digestibility (p < 0.05) and reducing digesta viscosity (p < 0.001). Furthermore, gut morphology was negatively affected by higher SFM inclusion, with significant reductions in VH and CD (p < 0.01), although NSPase supplementation helped maintain intestinal integrity.

One of the key strengths of this study is its controlled experimental design, which allowed for a rigorous evaluation of the effects of SFM inclusion and NSPase supplementation using a 3 × 2 factorial approach. The study also incorporated multiple response variables, including growth performance, nutrient digestibility, gut morphology, and digesta viscosity, providing a comprehensive understanding of dietary modifications in broiler nutrition. In addition, the findings offer practical implications for poultry nutritionists and feed manufacturers, suggesting that SFM can serve as a cost-effective alternative to SBM in regions where SBM is expensive or imported.

Despite these strengths, the study has certain limitations. The experiment was conducted under controlled conditions, which may not fully replicate commercial broiler production systems with varying management practices, environmental factors, and feed ingredient variability. In addition, while NSPase supplementation improved fiber digestibility and reduced gut viscosity, the specific enzymatic activity on different NSP components was not analyzed, which could provide further insights into enzyme efficiency.

Future research should focus on evaluating the long-term effects of SFM inclusion beyond the broiler phase, particularly its impact on carcass yield, meat quality, and immune function. In addition, further investigations are needed to optimize enzyme formulations by assessing the efficacy of different multi-enzyme complexes in breaking down NSPs. Metagenomic and microbiome studies could also provide deeper insights into the gut microbial response to high-fiber diets supplemented with NSPase. Moreover, economic feasibility studies comparing the cost-effectiveness of SBM replacement with SFM in different market conditions would help validate its commercial applicability. Overall, the study provides valuable evidence supporting the partial replacement of SBM with SFM (up to 20%) in broiler diets, particularly when supplemented with NSPase enzymes, thereby offering a sustainable and economically viable feeding strategy for the poultry industry.

## AUTHORS’ CONTRIBUTIONS

WA and MAUR: Conceptualization and writing–review and editing. ZM, SA, UF, and RM: Methodology. FR, WA, MR, and SH: Software. AR, MFK, WA, and MAUR: Validation. FR, MR, and MM: Formal analysis. ZM, SA, and UA: Investigation. WA, MFK, MAUR: Resources. ZM, SH, RM, and UF: Data curation. ZM and WA: Writing-original draft preparation. UA, SH, and SA: Visualization. WA: Supervision and project administration. All authors have read and approved the final manuscript.
